# Rapamycin Alleviates 2,4,6-Trinitrobenzene Sulfonic Acid-Induced Colitis through Autophagy Induction and NF-*κ*B Pathway Inhibition in Mice

**DOI:** 10.1155/2022/2923216

**Published:** 2022-08-18

**Authors:** Zhen Ni, Hao Li, Dong Mu, Juanni Hou, Xiaoyan Liu, Shanhong Tang, Shumei Zheng

**Affiliations:** Department of Gastroenterology, The General Hospital of Western Theater Command, Chengdu, Sichuan, China

## Abstract

**Background:**

Recent genetic studies indicated that variants of autophagy genes were associated with the predisposition of Crohn's disease (CD). The autophagy deficiency may affect the innate and adaptive immunity, which is related to persistent and excessive inflammation of the bowel. However, it remains unclear how autophagy modulates the expression of immune response regulator NF-*κ*B and proinflammatory cytokine TNF-*α* in CD.

**Aim:**

We aimed to investigate the role of rapamycin on the expression of NF-*κ*B p65 and TNF-*α* in 2, 4, 6-trinitrobenzene sulfonic acid (TNBS)-induced mouse colitis and lipopolysaccharide (LPS)-induced HT-29 cells.

**Methods:**

TNBS-induced colitis mice were treated with saline or rapamycin, and the disease activity index (DAI) and histological scores of colonic mucosa were evaluated. The expressions of p65, ATG16L1 and LC3 were detected by western blot and immunohistochemistry staining. The monodansylcadaverine (MDC) staining and transmission electron microscopy were developed to study the autophagy in LPS-induced HT-29 cells. Expression of TNF-*α* from colon tissue and HT-29 cells were detected by ELISA. The expressions of p65, ATG16L1 and LC3 in active CD patients were also investigated.

**Results:**

Significantly more autophagosomes were observed in rapamycin-treated cells than in controls. Rapamycin remarkably upregulated the expression of ATG16L1 and LC3II, inhibited p65 nucleus translocation and secretion of TNF-*α* both *in vivo* and *in vitro*. The expression of both ATG16L1 and LC3II increased in mild to moderate CD specimens, while no significant difference was noted between severe CD and normal controls. The expression of p65 increased notably in severe CD compared to those in mild to moderate patients.

**Conclusions:**

In LPS-treated HT-29 cells and TNBS-induced colitis, p65 is overexpressed, which results in exaggerated secretion of TNF-*α* and induce or worsen the inflammation in the bowel. Rapamycin protects against colitis through induction of autophagy, thus inhibiting the activation of NF-*κ*B pathway and secretion of TNF-*α*.

## 1. Introduction

Inflammatory bowel diseases (IBD), including CD and ulcerative colitis (UC), are chronic disorders characterized by excessive and/or sustained inflammation of the gastrointestinal tract [1]. The etiology of IBD remains unclear. The current opinion on pathogenesis of IBD has involved environmental factors, infectious agents, and genetic susceptibility, leading to abnormal mucosal immune response [1]. The main medical treatments for IBD are corticosteroids, immune suppressants such as azathioprine or 6-mercaptopurine, and biological agents such as anti-TNF-*α* antibody or anti-integrin antibody in severely active diseases or frequent relapse patients [2]. Although substantial numbers of the patients maintain remission, some cannot benefit from these medical therapies.

Recent genome-wide association studies have revealed that single-nucleotide polymorphisms in autophagy-associated genes, including ATG16L1 (autophagy-related 16 like 1) *^T300A^*, IRGM (immunity related GTPase M), and NOD2 (nucleotide-binding oligomerization domain containing 2) *^L1007fsinsC^* variants, are risk factors for CD [3]. Autophagy is an intracellular degradation pathway that regulates the turnover of cellular proteins and organelles and plays an essential role in cellular homeostasis [4]. In the intestine, autophagy has been reported to mediate critical functions in innate and adaptive immunity, such as antigen presentation by dendritic cells, cytokine secretion by macrophages, and antimicrobial peptide secretion by Paneth cells [5].

A large amount of evidences have demonstrated the crosstalk between autophagy and inflammation during pathophysiological situations. On one hand, autophagy modulates the inflammatory response through several mechanisms, including the selective degradation of both proinflammatory complexes, such as the NF-*κ*B essential modulator complex (NEMO), or inflammasome, and mitochondrial ROS or DNA [6]. On the other hand, autophagy is induced during inflammation by the activation of damage-associated molecular patterns (DAMPs) and several proinflammatory cytokines for controlling infection as part of the host response to microbial invasion [6]. Rapamycin, a widely used autophagy inducer, could downregulate the inflammatory processes and indicate its potential application in treating IBD [7].

In the present study, we investigated the role of rapamycin on the expression of NF-*κ*B p65 and TNF-*α* in TNBS-induced mouse colitis and LPS-induced HT-29 cells. The underlying mechanisms involved in the interaction existing between autophagy and immune response may provide a novel treatment for CD.

## 2. Material and Methods

### 2.1. Patients

Thirty patients, who were treated at the General Hospital of Western Theater Command between July 2016 and July 2021, include 15 healthy examination cases and 15 patients diagnosed with active CD, of which 7 were mild to moderate cases and 8 were severe. Intestinal tissue specimens were obtained for strict inclusion and exclusion criteria, and the use of glucocorticoid, immune inhibitors, and biological agents within three months should be excluded, which could affect the immune function. Crohn's disease activity index (CDAI) system [8] and simple endoscopic score for Crohn's disease (SES-CD) [9] were used for the assessment of CD scores. The study was approved by the Ethics Committee of the General Hospital of Western Theater Command (2021EC4-82).

### 2.2. Cell Culture and Treatment

HT-29 cells (ATCC, Manassas, VA, USA) were cultured in DMEM medium supplemented with 10% fetal bovine serum (HyClone, Logan, UT, USA) containing penicillin (100 U/ml) and streptomycin (100 U/ml) and incubated in a humidified incubator (95% air and 5% CO_2_) at 37°C. Rapamycin (Sigma-Aldrich, St. Louis, MO, USA) was dissolved in dimethyl sulfoxide (DMSO, Sigma-Aldrich, St. Louis, MO, USA). For LPS treatment, the cells were seeded into 6-cell plate culture dishes. When the confluence reached 60-70%, the cells were starved for 24 h and then treated with LPS (100 ng/ml, Sigma-Aldrich, St. Louis, MO, USA) or DMSO for 24 h in medium without fetal bovine serum. For pharmacological intervention, the cells were pretreated with rapamycin (25 *μ*g/ml) for 1 h and followed by LPS (100 ng/ml) or DMSO for 24 h. All assays were performed in triplicate.

### 2.3. Monodansylcadaverine Staining

Following rapamycin or LPS treatment, HT-29 cells were labeled with 0.05 mM MDC (Sigma-Aldrich, St. Louis, MO, USA) in phosphate-buffered saline (PBS) at 37°C for 10 minutes and washed with PBS for four times. Fluorescence slides were photographed immediately using a fluorescence microscope (Nikon Microscope, Tokyo, Japan) at a wavelength of 450-490 nm.

### 2.4. Transmission Electron Microscope

HT-29 cells were fixed in 2.5% glutaraldehyde for 30 min at 4°C. The fixed cells were washed by PBS gently and then postfixed in 1% osmium tetroxide for 30 min at room temperature. After postfixation, the samples were dehydrated in graded alcohol and processed for spur embedding. Ultrathin sections were cut on the Nova Ultratome (LKB Instruments, Broma, Sweden), collected on copper grids, and stained with uranyl acetate and lead citrate. Sections were observed under the transmission electron microscope (JEOL-1011; JEOL, Tokyo, Japan) at 60 kV.

### 2.5. TNBS-Induced Animal Models

Female BALB/c mice (15-22 g, 6-8 weeks old, China) were fed in Experimental Animal Center, the General Hospital of Western Theater Command. After 24 hours of fasting, TNBS colitis was induced as described previously [10, 11]. Briefly, mice were anaesthetized by intraperitoneal injection with 3% pelltobarbitalum natricum. 100 *μ*l of 2.0% (wt/vol) TNBS (Sigma-Aldrich, St. Louis, MO, USA) in 50% ethanol was slowly administered into the lumen of the colon via a 3.5 F catheter. Vehicle-treated mice were administered with 100 *μ*l of 50% ethanol. Animals were then kept in a vertical position for 30 s and returned to their cages. Two hours later, rapamycin (Sirolimos, 3 mg/kg/day) gavage was administrated every day for 7 days. 100 *μ*l of normal saline was gavaged as the positive control. The DAI was determined by scoring changes in weight, hemoccult positivity, or gross blood and stool consistency [12]. Mice were sacrificed by cervical dislocation 7 days after colitis induction. The study was approved by the Animal Care and Use Committee at the General Hospital of Western Theater Command (2021EC4-82).

### 2.6. Hematoxylin and Eosin Staining

Colon samples were fixed and embedded in paraffin and then stained with Hematoxylin and Eosin (H&E). The degree of inflammation on microscopic cross-sections of the colon was graded in a semiquantitatively method from 0 to 4 (0, no inflammation; 1, low level of inflammation, with scattered infiltrating mononuclear cells (1–2 foci); 2, moderate inflammation, with multiple foci; 3, high level of inflammation, with increased vascular density and marked wall thickening; 4, maximal severity of inflammation, with transmural leukocyte infiltration and loss of goblet cells) [13].

### 2.7. Immunohistochemistry (IHC) Staining

The colon tissue was fixed, embedded in paraffin, and sectioned 5 *μ*m as previously described. Briefly, colon tissue sections were incubated with primary antibody (LC3B 1: 400, Abcam; ATG16L1 1 : 100, Abgent; p65 1 : 200, Santa-Cruz) overnight at 4°C in the humidified chamber. Slides were visualized by DAB treatment and then scanned by Pannoramic Viewer (3DHISTECH, Ltd., Budapest, Hungary). Six high power fields were randomly captured, and two independent pathologists were invited to judge the slides. Then, positive cells and total cells in each field were counted, and the percentage of positive cells was calculated per slide.

### 2.8. Western Blots

Tissues and cells were placed in ice-cold lysis buffer (50 mM Tris, 5 mM EDTA, 250 mM NaCl, 0.1% Triton X-100, 1 mM phenylmethylsulfonyl fluoride, 5 *μ*g/ml aprotinin, 5 *μ*g/ml leupeptin, and 1 mM sodium orthovanadate), homogenized, and incubated in ice for 20 min. Samples were centrifuged at 11,000 g for 10 min, supernatants were collected, and the protein concentration was measured with a BCA Protein Assay Kit (Pierce). Equal amounts of protein from colonic tissues or cell lines were loaded onto SDS-PAGE gels. After electrophoresis and transference, membranes were blocked with 5% nonfat dry milk in TBST and incubated overnight at 4°C with primary antibodies (LC3B 1 : 400, Abcam; ATG16L1 1 : 100, Abgent; p65 1 : 200, Santa-Cruze; *β*-actin 1 : 2000, Abcam; GAPDH 1 : 2000, Abcam). Subsequently, membranes were incubated with secondary antibody (anti-rabbit or anti-mouse IgG, 1 : 2000, Abcam). Signals were detected using WesternLumaxLight Sirius HRP substrate reagent (ZETA-Life, San Francisco, CA, USA). The bands were scanned using a ChemiDocXRS+Imaging System (Bio-Rad, Hercules, CA, USA) and quantified by Image Lab v5.2 software (Bio-Rad).

### 2.9. ELISA Assays

Colon tissue per gram was homogenized in 4 ml PBS, and then, the supernatant was collected after centrifugation. TNF-*α* in cell culture supernatant and colonic tissue homogenate was measured by using DuoSet ELISA development kits (R&D systems, Minneapolis, MN) according to the manufacturer's instructions.

### 2.10. Statistical Analysis

All cell culture experiments were performed for three times. Data were expressed as the means ± SEM. Difference between multiple groups was compared by one-way analysis of variance followed by Dunnett's post hoc tests. Statistical analysis was performed by SPSS 13.0 software (SPSS, Inc., Chicago, IL, USA) or GraphPad Prism 5.0 (GraphPad Software, San Diego, CA, USA). *P* < 0.05 was considered statistically significant.

## 3. Results

### 3.1. Rapamycin Protects against TNBS-Induced Colitis in Mice

**Figure 1:**
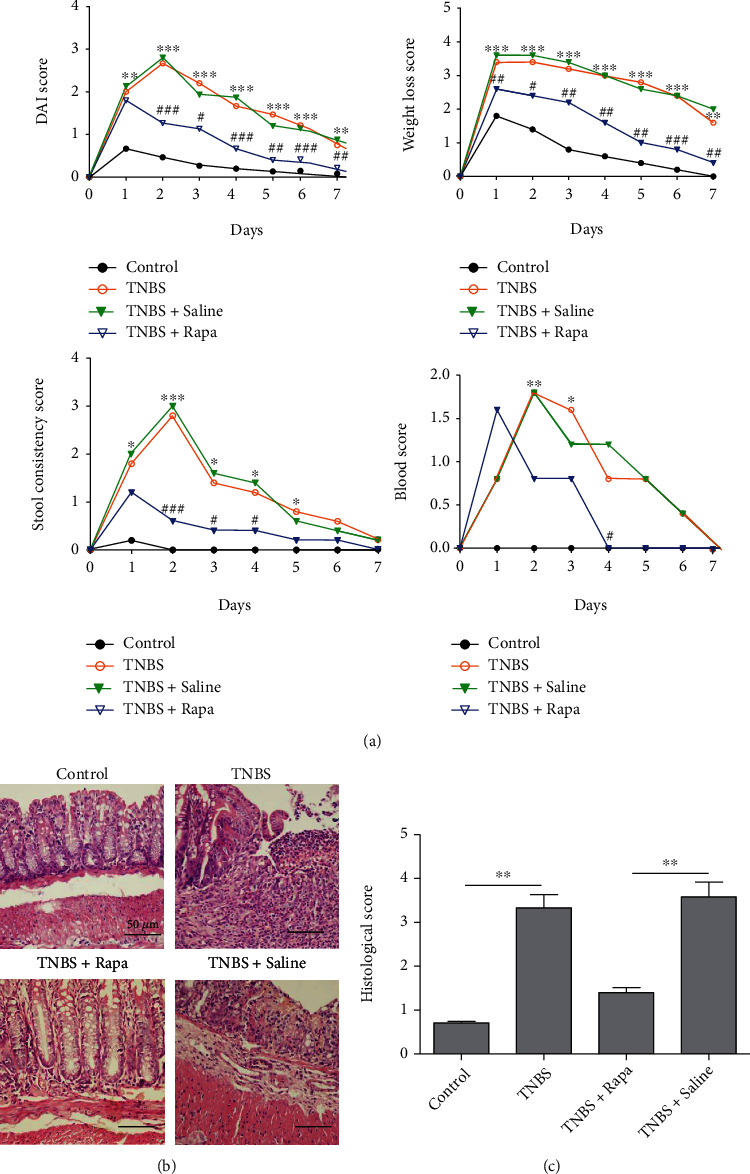
Rapamycin protects against TNBS-induced colitis in mice. (a) After being treated with TNBS (100 *μ*l of 2.0%), mice were gavaged with rapamycin (3 mg/kg/day) or saline (100 *μ*l) for 7 days. Disease activity index (DAI) scores were used to evaluate the severity of colitis. The DAI was determined by scoring changes in weight, hemoccult positivity, or blood trace and stool consistency. ^∗^TNBS *vs.* control; ^#^TNBS+Rapa *vs.* TNBS+saline. ^∗∗^*P* < 0.01,  ^∗∗∗^*P* < 0.001,  ^#^*P* < 0.05,  ^##^*P* < 0.01, and^###^*P* < 0.001. (b) H&E staining was used to examine the histological changes of colonic mucosa (×100). (c) The histological scores were calculated to evaluate the severity of colonic mucosa inflammation (*n* = 5 per group, ^∗∗^*P* < 0.01).

Among various animal models, TNBS-induced colitis has been broadly used to study a variety of aspects relevant to CD due to its immunological and histopathological characteristics. We used DAI score to evaluate the severity of TNBS-induced colitis in mice. As shown in Figure 1(a), significant higher DAI scores including body weight loss, stool consistency, and blood were observed in TNBS-treated groups than in control mice after the second day of TNBS administration. Rapamycin gavage administration, however, decreased the DAI scores from the second day of the TNBS-treated mice. On day 8, the DAI score was significantly lower in the rapamycin treatment group than in the TNBS group. No significant difference of DAI score was noted between rapamycin-treated mice and control mice on day 8 after TNBS treatment.

To evaluate the histological findings of these animals, we then examined the colitis severity based on the inflammation score using H&E staining. The results showed that TNBS induced strong transmural inflammation, ulceration, and loss of crypt structure and goblet cells, while rapamycin significantly improved the inflammation, with only occasional small inflammatory infiltration compared with the TNBS group (Figure 1(b)). The microscopic score of the TNBS-treated group increased significantly than control mice. In contrast, rapamycin significantly decreased TNBS-induced inflammation scores (Figure 1(c)).

### 3.2. Rapamycin Induces Autophagy In Vivo

The induction of autophagy was assessed by detecting an increase of the LC3, which is considered as a specific marker of autophagy. There are two cellular forms of the LC3 protein. One is LC3I, a cytoplasmic form of LC3, and another one is LC3II, a cleavage form of LC3, which is associated with the autophagosomal membrane. Thus, the increased ratio between LC3II and LC3I is associated with autophagy induction. ATG16L1 is another maker to monitor autophagy. Here, we detected the expression autophagy markers, LC3 and ATG16L1, by both IHC staining and western blot.

**Figure 2:**
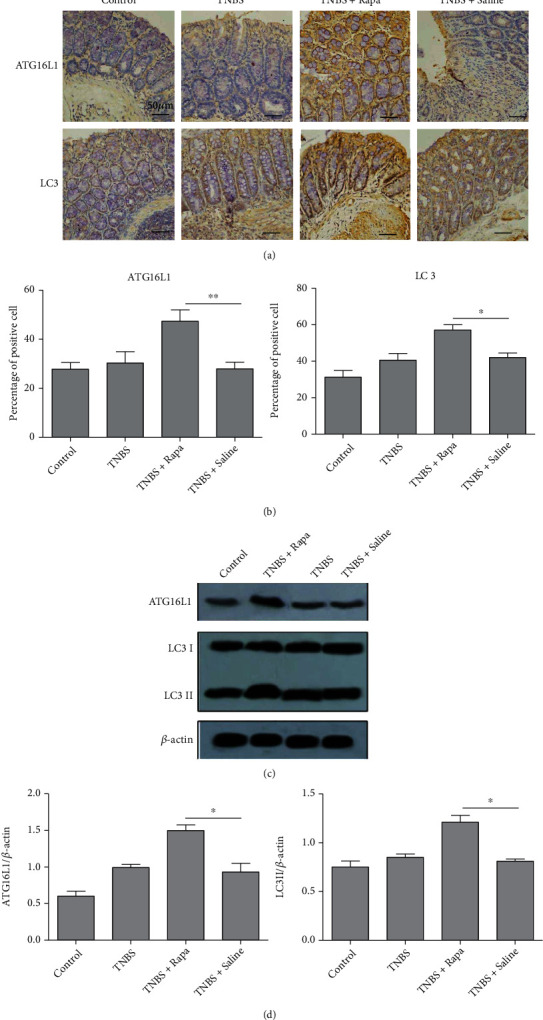
Rapamycin induces autophagy *in vivo.* Mice were treated as the same in Figure 1 for 7 days. (a) The expression of colonic ATG16L1 and LC3 was detected using IHC. (b) The percentage of ATG16L1 or LC3-positive cells was demonstrated. (c, d) The expression of ATG16L1, LC3I, and LC3II in colonic tissues was measured using western blot. *n* = 5 per group; ^∗^*P* < 0.05 and^∗∗^*P* < 0.01.

The IHC result showed that ATG16L1 was mainly expressed in the cytoplasm or membrane of intestinal epithelial cells or partially expressed in lamina propria mononuclear cells, while LC3 was mainly expressed in the cytoplasm of these cells (Figure 2(a)). The percentage of ATG16L1 or LC3-positive cells increased significantly in colitis tissues of rapamycin-treated mice than TNBS-treated mice and control mice (Figure 2(b)). We subsequently detected the ATG16L1 and LC3II expression by western blot. The results showed that rapamycin significantly increased the expression of both ATG16L1 and LC3II in mice (Figures 2(c) and 2(d)), suggesting the induction of autophagy in the colonic tissues of rapamycin-treated animals.

### 3.3. Rapamycin Induces Autophagy In Vitro

**Figure 3:**
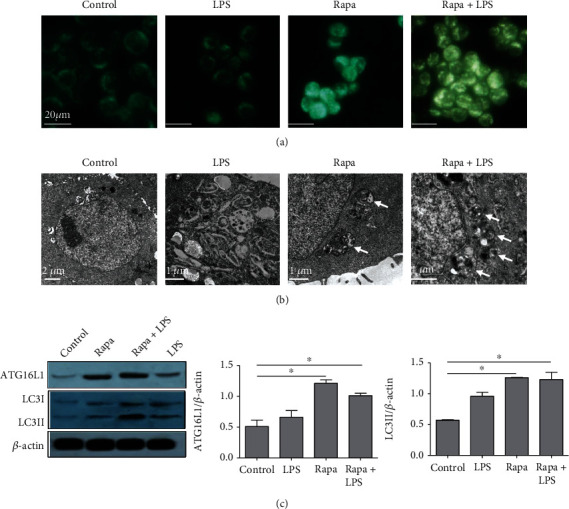
Rapamycin induces autophagy *in vitro*. HT-29 cells were stimulated by LPS (100 ng/ml) with or without rapamycin (25 *μ*g/ml) pretreatment for 1 h. (a) The formation of autophagic corpuscles in HT-29 cells were detected by MDC. (b) The intracellular autophagosomes were observed using electron microscopy (arrow). (c) The expression of ATG16L1, LC3I, and LC3II in HT-29 cells was detected by western blot *n* = 3 per group; ^∗^*P* < 0.05.

The unique feature of autophagy to engulf part of cytoplasm is the double-membrane structures. Our subsequent efforts were directed to determine whether rapamycin induces autophagy in LPS-stimulated HT-29 cells, an inflammatory intestinal epithelial cell model. Firstly, the formation of autophagic corpuscles was detected by MDC staining in HT-29 cells stimulated by LPS with or without rapamycin pretreatment. The results indicated that significantly more fluorescence aggregation points were observed in cells induced by 25 *μ*g/ml rapamycin for 24 h, while a few fluorescence aggregation points were observed either in LPS-treated cells or in control group, suggesting that cell autophagy was evidently induced by rapamycin (Figure 3(a)).

To better investigate the induction of autophagy in subcellular level, we then use electron microscopy to observe the intracellular autophagosomes in HT-29 cells. As expected, a few autophagosomes were observed in either vehicle-treated cells or LPS-stimulated cells, as shown in Figure 3(b); however, the exposure of HT-29 cells to rapamycin greatly increased the number of autophagosomes in the cytoplasm, suggesting the induction of autophagy by rapamycin.

Next, we evaluated the induction of autophagy in molecular level by detecting the expression of both ATG16L1 and LC3 in HT-29 cells. The western blot results revealed that rapamycin significantly increased LC3II levels, leading to the increased ratio of LC3II/LC3I in HT-29 cells (Figure 3(c)). Consistently, the expression of ATG16L1 was significantly elevated after rapamycin treatment (Figure 3(c)).

### 3.4. Rapamycin Inhibits NF-*κ*B p65 Nucleus Translocation and TNF-*α* Secretion Both In Vivo and In Vitro

Previous studies indicated that the activation of NF-*κ*B pathway led to the nuclear translocation of p65 and subsequent transcription of proinflammatory cytokines, such as TNF-*α*. When activated by LPS, intestinal epithelial cells secreted proinflammatory cytokines, including TNF-*α*, a key cytokine in IBD. We next investigated the effects of rapamycin on p65 expression and TNF-*α* production in both TNBS-induced colitis and LPS-stimulated HT-29 cells.

**Figure 4:**
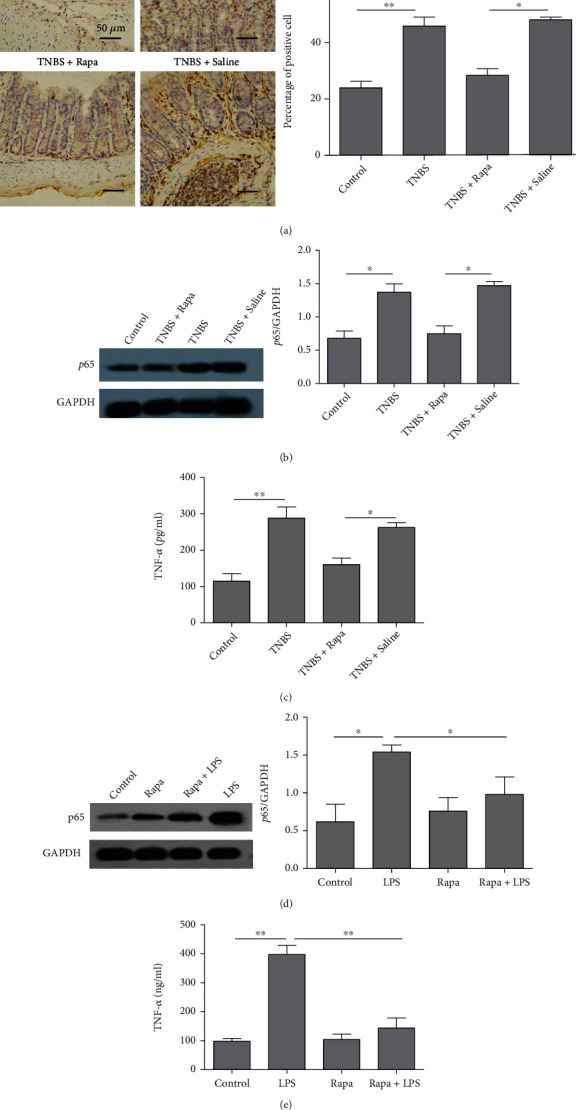
Rapamycin inhibits p65 nucleus translocation and TNF-*α* secretion both *in vivo* and *in vitro*. Mice were treated as the same in Figure 1 for 7 days. (a) The expression of p65 in colon tissue was detected using IHC staining (×100). (b) The expression of p65 in colon tissue was detected using western blot. (c) The expression of TNF-*α* from colon tissue was detected by ELISA (*n* = 5 per group). (d) HT-29 cells were stimulated by LPS (100 ng/ml) with or without rapamycin (25 *μ*g/ml) pretreatment for 1 h. The expression of p65 was detected by western blot. (e) The expression of TNF-*α* from HT-29 cells was detected by ELISA. *n* = 3 per group; ^∗^*P* < 0.05 and^∗∗^*P* < 0.01.

To investigate the expression of p65 and TNF-*α in vivo*, colonic tissues of the TNBS-treated mice were collected. Both IHC and western blot results indicated that TNBS significantly increased the expression of p65 in colitis tissue, which was significantly inhibited by rapamycin treatment (Figures 4(a) and 4(b)). Furthermore, TNF-*α* secretion by intestinal mucosa was obviously enhanced in TNBS groups, while rapamycin greatly decreased the TNF-*α* level in the intestinal mucosa (Figure 4(c)). The above results demonstrated that rapamycin may inhibit the nuclear translocation of p65 proteins and TNF-*α* secretion.

We subsequently detected the expression of p65 and TNF-*α in vitro.* HT-29 cells were pretreated with rapamycin for 1 h and subsequently stimulated with LPS for 24 h. The results indicated that LPS treatment significantly promoted p65 translocation into nucleus, while rapamycin markedly inhibited the p65 translocation induced by LPS (Figure 4(d)). In addition, ELISA assay showed that TNF-*α* secretion by HT-29 cells was significantly enhanced after incubation with LPS, while rapamycin dramatically inhibited the LPS-induced TNF-*α* secretion (Figure 4(e)).

### 3.5. The Expression of Autophagy Markers and NF-*κ*B p65 in Colitis Tissues of CD Patients

**Figure 5:**
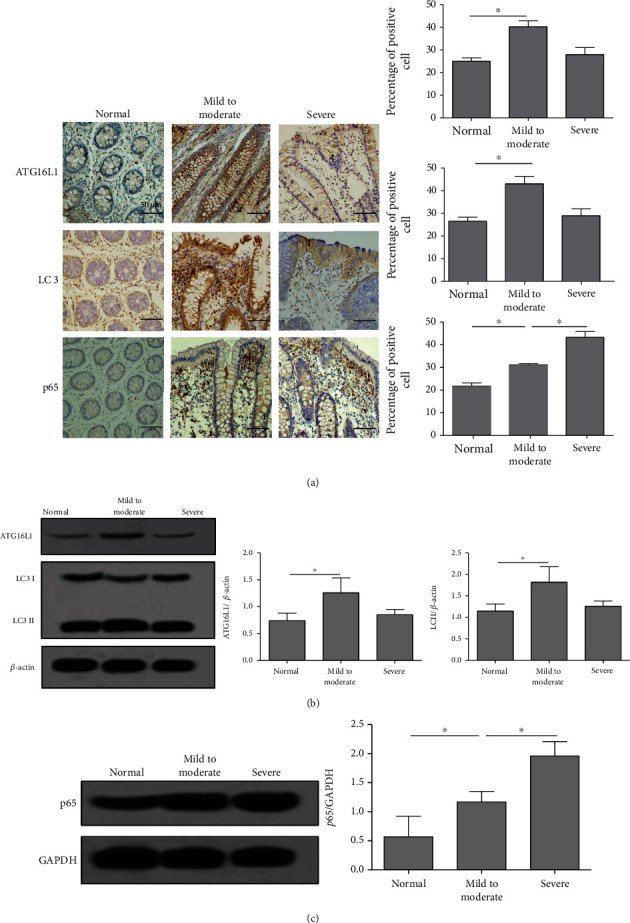
The expression of autophagy markers and p65 in colonic tissues of CD patients. (a) The percentage of ATG16L1, LC3, and p65 in colonic mucosa of normal controls or CD patients was detected using IHC staining (×100). (b) The expression of ATG16L1, LC3, and LC3II in colon tissues of normal controls or CD patients was detected using western blot. (c) The expression of p65 in colon tissues of normal controls or CD patients was examined by western blot. ^∗^*P* < 0.05.

To investigate the induction of autophagy and the activation of NF-*κ*B in CD patients, we then detected the expression of ATG16L1, LC3, and p65 in endoscopic mucosal biopsy specimens from 15 cases of active CD subjects and 15 cases of normal control ileocolonic tissues by IHC and western blot methods. The IHC results showed that ATG16L1 was mostly expressed in the cytoplasm and membrane of colonic epithelial cells and partially expressed in mononuclear cells, while LC3 was mainly expressed in the cytoplasm of mononuclear cells (Figure 5(a)). The p65 was mainly expressed in the nucleus of mononuclear cells and partially in colonic epithelial cells (Figure 5(a)). Data from western blot assay showed that expressions of ATG16L1 and LC3 II, two indicators of autophagy, were increased in mild to moderate CD specimens, while no significant differences were noted between severe CD and normal controls (Figure 5(b)), suggesting a relative higher activated autophagy in mild to moderate CD and a relative low autophagic activation in severe patients. In contrast, the expression of p65 notedly increased in severe CD compared to mild to moderate CD (Figure 5(c)), which were consistent with the results of IHC. These results indicated that the activation of NF-*κ*B significantly elevated in colonic tissues of severe CD, while the induction of autophagy increased insignificantly in these cases.

## 4. Discussion

Autophagy, a physiological self-protection process, plays a key role in cellular housekeeping. It is a highly sensitive process that cells are induced in response to various stressful conditions such as physical, chemical, biological, or metabolic stimuli in order to maintain cellular homeostasis [14]. Autophagy is crucial in regulating the interaction between gut microbiota and innate and adaptive immunity, in controlling mucosal inflammation and in maintaining intestinal homeostasis [15, 16]. A dysfunction of autophagy leads to the disruption of mucosal immunity in both cell-autonomous and cell nonautonomous manners, such as antigen processing and presentation, and the clearance of intracellular bacteria [17]. Furthermore, autophagy also plays an important role in the limitation of proinflammatory Th2 cell expansion and in the promotion of regulatory T cell survival. Gutierrez et al. reported that physiological induction of autophagy or its pharmacological stimulation by rapamycin suppressed intracellular survival of mycobacteria [18]. Previous studies showed that following stimulation with LPS, Atg16L1-deficient macrophages produce high amounts of inflammatory cytokines IL-1*β* and IL-18 [19, 20]. They subsequently found that mice lacking Atg16L1 in hematopoietic cells are highly susceptible to dextran sulfate sodium- (DSS-) induced acute colitis [21, 22]. Our present study indicates that rapamycin-induced autophagy significantly alleviates experimental colitis and decreases the TNF-*α* secretion from LPS-induced HT-29 cells, which were in accordance with the previous studies. These results suggest that regulation of autophagy may be a therapeutic target for damping inflammatory response.

Rapamycin, a calcineurin inhibitor, similar to cyclosporine A (CsA) and FK506, has been widely used in preventing allograft rejection [23]. It exerts an effect by binding to the intracellular immunophilin FK506 binding protein (FKBP12) [24]. However, unlike CsA and FK506, the rapamycin-FKBP12 complex inhibits the function of mammalian target of rapamycin (mTOR), which in turn reduces protein phosphorylation and inhibits the cell cycle [25]. Rapamycin markedly decreases the number of lymphocytes, including CD4+ subsets but increases the percentage of immunosuppressive CD4+/CD25+ regulatory T cells (Tregs) [26, 27]. Owing to its different effects on Tregs and effector T cells, rapamycin has been used in posttransplantation management and is expected to be used in T cell-mediated disorders, including IBD [7, 28]. However, the underlying mechanism involved in how rapamycin suppresses experimental colitis remains unclear. Farkas et al. reported that sirolimus, an analogue of rapamycin, by decreasing leukocyte migration, is as effective as CsA in DSS-induced colitis [29]. Matsuda et al. reported that everolimus, another structural analogue of rapamycin, ameliorates an IL10^–/–^ colitis in mice probably as a result of decreasing the number of CD4+ T cells in the colonic mucosa and an associated reduction of IFN-*γ* production [30]. Zhao et al. demonstrated the protective effect of rapamycin in IL-10^−/−^ mice through induction of autophagy, which significantly improved intestinal barrier function and decreased proinflammatory cytokines [31]. Similarly, our study also indicated that rapamycin ameliorated inflammation through inhibition of the NF-*κ*B pathway in TNBS-induced colitis.

NF-*κ*B, a crucial transcription factor involved in inflammation, may promote inflammation response through regulating expression of downstream inflammation-related genes, such as inducible nitric oxide synthase (iNOS), TNF-*α*, and IL-1*β* [32]. NF-*κ*B consists of five family members in mammalian cells, including p50, p52, RelA (p65), c-Rel, and RelB [32]. The most abundant form of NF-*κ*B is a heterodimer of the p65 and p50. In most cell types, this complex normally exists in an inactive state bound to a family of I*κ*B inhibitory proteins in the cytoplasm. Once I*κ*B is phosphorylated in response to some specific stimuli, p65 is released to translocate freely to the nucleus, where it can activate the transcription of target genes such as some proinflammatory cytokines [33]. Inhibition of NF-*κ*B signaling has been demonstrated to ameliorate inflammation. Increasing evidences indicate that rapamycin-induced autophagy exhibits anti-inflammatory actions through inhibiting the activation of NF-*κ*B by selective degradation of NF-*κ*B-inducing kinase (NIK) and I*κ*B kinase (IKK), two key kinases in the NF-*κ*B activation [25, 34]. Our data showed that rapamycin suppressed LPS-induced upregulation of p65 translocation, thus inhibiting the secretion of TNF-*α*, a key proinflammatory cytokine in IBD. However, conflicting data have been published pertaining to the inhibitory or stimulatory effects of rapamycin on NF-*κ*B activity, which might be due to different cells models [35–37].

As reported previously, treatment with rapamycin ameliorates CD-like ileitis in animal models [30, 31] and improves symptoms in CD patients [38, 39]. The mechanism involved in the relation between autophagy and CD is not fully understood. In our study, we investigated the role of rapamycin in both TNBS-induced mice and LPS-induced HT-29 cells and found that rapamycin reduced the expression of p65 and TNF-*α* through induction of autophagy. Moreover, we further validated the expression of ATG16L1, LC3, and p65 in CD patients and the conflicting expression patterns in severe CD subgroup suggesting a potential therapy for severe CD patients. In mild to moderate CD cases, autophagy of intestinal mucosa was significantly induced, which could be reinforced in TNBS-induced mice and LPS-induced HT-29 cells. However, insufficient autophagy in severe CD patients contributed to the burst of p65, which might be the underlying mechanism of CD progress. Therefore, manipulation of autophagy may have therapeutic merit for patients affected by severe CD.

The mechanisms of crosstalk between autophagy and inflammation and how autophagy shapes the inflammatory response are still poorly understood. In light of studies during the last decade, autophagy contributes to damp inflammatory responses by at least two means to protect cells from excessive long-lasting inflammation: indirectly by clearance of damaged organelles or intracellular pathogenic microorganisms and directly by suppressing proinflammatory complexes [6, 40]. The results of the present study demonstrate that rapamycin blocks LPS-induced HT-29 cell inflammation and TNBS-induced colitis. The anti-inflammatory mechanism of rapamycin may be its induction of autophagy and thereby the inhibition of NF-*κ*B activation. Our findings provide theoretical evidence for rapamycin as a candidate for the treatment of severe CD subgroup. Further clinical studies are required to validate the application of rapamycin in these diseases.

## 5. Conclusions

In LPS-treated HT-29 cells and TNBS-induced colitis, p65 is overexpressed, which results in exaggerated secretion of TNF-*α* and induce or worsen the inflammation in the bowel. Rapamycin protects against colitis through induction of autophagy, thus inhibiting the activation of NF-*κ*B pathway and secretion of TNF-*α*. The effect of rapamycin in human subjects remained to be further evaluated in an approved clinical trial.

## Data Availability

The data that support the findings of this study are available from the corresponding author, Dr. Shumei Zheng, upon reasonable request.
